# Conformation-Dependent Lesion Bypass and Mutagenicity of Bulky 2‑Acetylaminofluorene-Guanine DNA Adduct in Epigenetically Relevant Sequence Contexts

**DOI:** 10.1021/acs.chemrestox.5c00055

**Published:** 2025-07-04

**Authors:** Yi-Tzai Chen, Rui Qi, Ang Cai, Bongsup P. Cho, Deyu Li

**Affiliations:** Department of Biomedical and Pharmaceutical Sciences, College of Pharmacy, 4260 University of Rhode Island, Kingston, Rhode Island 02881, United States of America

## Abstract

DNA base cytosine can be modified epigenetically by adding a methyl group to form 5-methylcytosine (5mC). 5mC in DNA CpG islands plays a crucial role in mammalian cell development and epigenetic regulation. While 5mC does not block DNA replication and is not mutagenic, the biological consequences of 5mC affecting the flanking guanine with a bulky modification during DNA replication are not well understood. This paper examined the lesion bypass and mutagenicity of the 2-acetylaminofluorene-modified guanine DNA adduct (dG-AAF) in epigenetically relevant sequence contexts in *Escherichia coli*. The C/5mC context exhibited significantly different bypass and mutagenicity profiles for dG-AAF. The biological outcomes also varied depending on the nature of the 3′ flanking base and the lesion bulkiness. In addition, we extensively observed a unique type of −1 G deletion when the lesion was flanked by 3′ purines, possibly due to the formation of a stacked slipped mutagenic intermediate. However, there was no such deletion with 3′ pyrimidines. Our findings provide a new perspective on the role of epigenetic markers in DNA replication and could help to develop methods to identify mutation patterns associated with specific mutational signatures or spectra in cancer.

## Graphical abstract

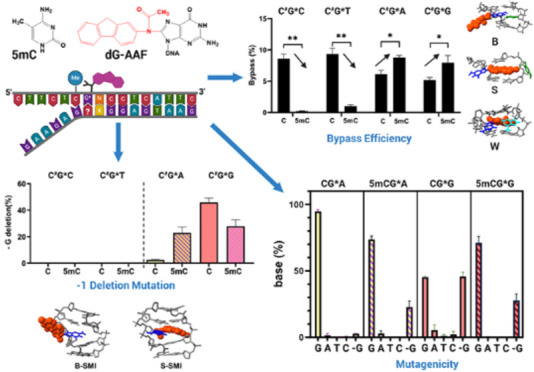

## Introduction

DNA base cytosine can be modified by adding a methyl group to its 5-position, forming 5-methylcytosine (5mC). The 5mC modification is the most common type of DNA methylation in mammals, and it mainly happens in sequences in which 5′ cytosine is followed by guanine (CpG sites). Special enzymes (S-adenosylmethionine-dependent methyltransferases) add the methyl group to form 5mC.^1,2^ There is also a pathway to reverse this process (demethylation). Ten-eleven translocation (TET) proteins, with their ability to iteratively oxidize the 5mC to 5-hydroxymethyl cytosine (5hmC), 5-formyl cytosine (5fC), and 5-carboxyl cytosine (5caC), play a crucial role in this reversal process.^3–5^ Then, thymine DNA glycosylase (TDG) removes 5fC and 5caC, turning 5mC back into cytosine through a base excision repair (BER) mechanism.^6–9^ 5mC and its oxidative modifications play critical roles in cell development and epigenetic regulations;^5,10^ however, malfunctions in this system have been linked to cancer and other diseases.^11–15^

While 5mC itself has been demonstrated to have a relatively minor effect on DNA replication and mutagenicity,^16,17^ its influence on DNA replication with neighboring damaged bases is not fully understood and remains a complex and intriguing research area. Watt et al. have shown that having 5mC instead of cytosine next to the bulky guanine modification (*N*-(deoxyguanosine-8-yl)-1-aminopyrene) in a p53 tumor suppressor gene doubled the mutation rate of the guanine.^18^ The results suggest a complex interplay between 5mC and the mutations of neighboring bases. Feng et al. revealed that methylation of CpG sites within the human p53 gene increases the DNA adduct formation caused by 4-aminobiphenyl (ABP) and creates newly recognized binding sites.^19^ In this case, the structural basis for 5mC’s increased lesion formation and mutations is unknown. The presence of 5mC instead of cytosine next to guanine increases the guanine’s chemical reactivity to form antibenzo­[*a*]­pyrene 7,8-dihydrodiol 9,10-epoxide (anti-BPDE) dG adducts.^20^ The data show that sequences containing 5mC exhibit increased thermal stability and conformational heterogeneity. NMR results reveal that 5mC affects the interaction of the two diastereomers of anti-BPDE with DNA differently. The 10R(−)-*trans* isomer, when bound next to an unmethylated cytosine (CpG), adopts a specific conformation where the planar aromatic rings are located within the minor groove of the DNA. However, when bound next to a 5mCpG, it changes to a base-displaced conformation where the BPDE moiety gets partially inserted between the DNA bases. The conformation of the 10S­(+) isomer remains unchanged regardless of CpG methylation, staying in the minor groove in both cases. The impact of these drastic conformational alterations on the processing of lesions by cellular repair and replication mechanisms remains unexplored. For example, the 5mC change from C affects the accessibility and recognition of lesions by repair enzymes or the bypass fidelity of DNA polymerases during replication.

Arylamines, environmental chemicals that can form bulky DNA adducts, are suspected to cause certain human cancers, particularly bladder and liver cancers.^21,22^ 2-Acetylaminofluorene (AAF) was initially developed as an insecticide for farms, but it was later withdrawn from the market due to its ability to cause tumor growth in rats’ livers.^23^ AAF has been extensively used as a model mutagen. Inside living organisms, AAF is known to metabolize to a highly electrophilic nitrenium ion and then interact with DNA, forming two primary bulky lesions on guanine: *N*-acetylated AAF (dG-AAF) or *N*-deacetylated 2-aminofluorene AF (dG-AF) adduct on the C8 position of guanine (Figure 1).^24,25^ 2-Nitrofluorene is an IARC-designated Group 2B carcinogen and an environmental pollutant that forms these two DNA adducts upon exposure.^26,27^ dG-AF is mainly nonmutagenic; in contrast, dG-AAF strongly blocks polymerases, resulting in frameshift and point mutations.^28,29^ These adducts are believed to form in both epigenetic and nonepigenetic sequence contexts with similar chemical reactivities.^30^

**Figure 1:**
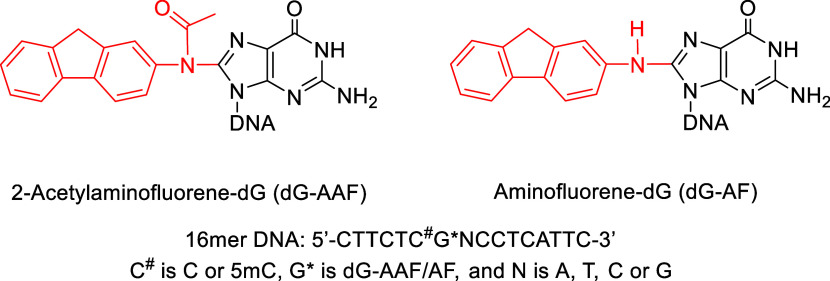
Chemical structures of dG-AAF and dG-AF and DNA sequences used in this study.

The *Nar*I recognition sequence (5′-G_1_G_2_CG_3_*CC-3′, where G_3_ is modified) is a mutational hotspot of dG-AAF. The G_3_ lesion in the *Nar*I region of DNA primarily causes a −2 frameshift deletion (resulting in the deletion of CG bases) during translesion synthesis (TLS).^31^ Although the dG-AAF lesions can occur at three guanine sites (G1, G2, and G3), the −2 deletion occurs only when the lesion is located at G_3_. ^19^F NMR data revealed a unique structure: the bulky dG-AAF lesion forces the guanine lesion into a *syn*-orientation, where the aromatic fluorene portion stacks with the neighboring base pairs. This structure facilitates the DNA replication machinery’s ability to slip, resulting in the exclusion of two bases (CG) during the copying process, which explains the −2 frameshift mutation.^32–34^

Previously, we studied the conformational effects of 5mC on the cellular bypass efficiency of the nonacetylated, less bulky *N*-(2′-deoxyguanosin-8-yl)-7-fluoro-2-aminofluorene lesion (dG-FAF). Combined with NMR data, we found conformation-dependent bypasses of dG-FAF in epigenetically relevant sequence contexts ([5′-C^#^G*N-3′], where G* is dG-FAF, C^#^ is C or 5mC, and N is A, T, C, or G). Sequences with a 3′ flanking pyrimidine next to the lesion are better bypassed when the 5′ base is 5mC instead of C. NMR observations rationalize the conformational basis behind these observations: for -C^#^G*T- and -C^#^G*C- (3′ flanking pyrimidine), the bypass efficiency appears to be inversely correlated with the stacked conformation population of dG-FAF. On the contrary, sequences with a 3′ flanking purine base exhibit the opposite effects.

This paper examined the biological consequences of bulkier dG-AAF during DNA replication in *E. coli*. We discovered a unique −1 deletion mutation when dG-AAF is present in the C^#^G*A and C^#^G*G sequences, but not in the C^#^G*C and C^#^G*T sequences. We also observed that the C/5mC context has very different profiles in terms of the bypass efficiency and mutagenicity of the dG-AAF lesion.

## Results

### Selection of Single-Stranded M13 Vector

To study the replication bypass and mutagenicity of dG-AAF adduct, we adopted the competitive replication of adduct bypass (CRAB) and restriction endonuclease and postlabeling (REAP) assays developed by the Essigmann lab,^35–37^ which used a single-stranded- (ss-) M13 vector. The reason for choosing an ss-DNA vector, such as the M13 vector used here, was to avoid the repair of dG-AAF adducts by the nucleotide excision repair (NER) enzymes.^38,39^ dG-AAF adduct has been reported to be repaired by transcription-coupled nucleotide excision repair (TC-NER) in double-stranded- (ds-) DNA.^40,41^ Thus, the cellular data could reflect the biological properties of the dG-AAF adduct itself without complications from repair by NER pathways.

### Selection of AlkB-Negative *E. coli* Strain

5mC has been reported to be oxidatively modified by the TET family enzymes (TET1 to 3) to h5mC, f5C, and ca5C in an iterative process.^5^ We also found that AlkB and its human homologues ALKBH2 and 3 can modify 5mC to those oxidative intermediates.^42^ There are nine AlkB homologues (ALKBH1 to 8 and FTO) in human cells. Enzymes from the AlkB and TET family use an α-ketoglutarate (αKG)/Fe­(II)-dependent mechanism to oxidize DNA/RNA substrates.^10^ Besides ALKBH2 and 3 and TET 1 to 3, other AlkB homologues could also potentially oxidize 5mC. To simplify the complications from those proteins, we selected *E. coli* cells because there is only one AlkB protein and no TET protein in *E. coli*. For the *E. coli* cell, AlkB- (HK82) strain^37^ was chosen to avoid AlkB’s oxidative modification of 5mC; even the dG-AAF adduct has not been reported as a substrate of AlkB. Additionally, various vector systems have been employed to investigate DNA adducts in both *E. coli* and human cells. The biological consequences (such as replication block and mutagenicity) generated from the two sources for an individual adduct are very similar.^43,44^ Even though 5mC does exist in *E. coli* cells, it does not occur at the CpG site but happens at the second cytosine in the sequence 5′ CCWGG 3′ (W = A/T), which is part of the *Eco*RII restriction modification system.^45^ Those observations and reasons validate the use of the HK82 *E. coli* strain to study the biological properties of a particular DNA adduct, such as the dG-AAF adduct tested in this paper. Furthermore, the AlkB-negative strain showed the expected replication block and mutation patterns of 1,*N* ^6^-ethenoadenine (εA),^36^ which was used as a positive control to validate the successful application of the CRAB and REAP methods (Figure S32). Future investigations of dG-AAF’s biological properties would be carried out in human cells with different AlkB homologues and TET proteins capacities.

### Lesion Bypass and Mutagenicity of dG-AAF in the Epigenetically Relevant Sequence Contexts

The lesion bypass (CRAB) assay exhibited clear and distinct trends in the bypass efficiencies of dG-AAF adducts depending on the 5mC/C sequences. The presence of a 5′ 5mC to the lesion, following a 3′ pyrimidine (C/T), significantly reduced bypass efficiency (Figure 2a). The efficiency dropped from 8.6% in CG*C to 0.2% in 5mCG*C (*P* < 0.01), and from 9.3% in CG*T to 1.0% in 5mCG*T (*P* < 0.01). Both situations (for 3′ C and T) represented 90% decrease in bypass efficiency by replacing C with 5mC. Conversely, when dG-AAF was followed by a 3′ purine base (A or G), the bypass efficiency of the lesion revealed an opposite trend. The bypass increased from 6.2% in CG*A to 8.8% in 5mCG*A (*P* < 0.05), and from 5.2% in CG*G to 7.9% in 5mCG*G (*P* < 0.05) (Figure 2a). Both situations (for 3′ A and G) represented a 40% increase in bypass efficiency by replacing C with 5mC. These findings presented clear and opposite trends for bypass efficiency of dG-AAF with C versus 5mC on the 5′ end, followed by either 3′ pyrimidines or 3′ purines.

**Figure 2:**
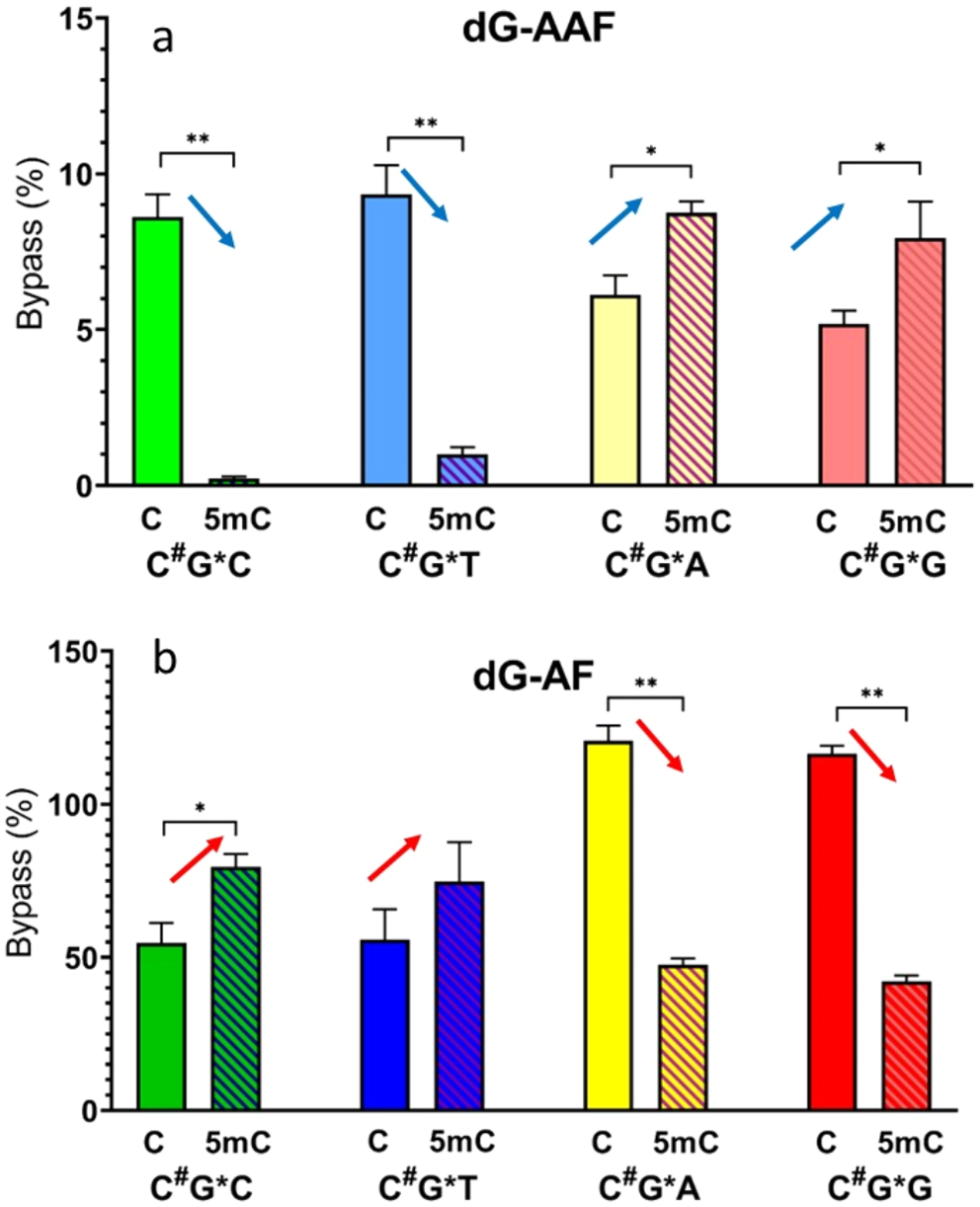
Bypass efficiencies of (a) dG-AAF and (b) dG-AF under different sequence contexts. C^#^ represents either C or 5mC and G* represents either dG-AAF or dG-AF. Data in panel b were extracted from our previously publication on dG-AF.^30^ Data are presented as the arithmetic mean (*n* = 3) ± standard deviation (error bar). Arrows indicate upward and downward trends. An unpaired Student’s *t*-test was used to assess statistical significance; *P* < 0.05 was considered significant. Statistical significance: **P < 0.01 and *P < 0.05. Note: Bypass >100% is common as bypass in the CRAB assay was calculated relative to the identical sequence containing an unmodified G.^46,47^

Previously, we studied the bypass of the less bulky dG-AF (using fluorine-labeled FAF) adduct using similar sequence contexts.^30^ The dG-AF results from the previous paper (Figure 2b) reveal patterns opposite those of dG-AAF presented above. The presence of a 5′ 5mC to dG-AF following a 3′ pyrimidine (C/T) enhances bypass efficiency (Figure 2b): efficiency increases from 54.7% in CG*C to 79.7% in 5mCG*C, and from 55.7% in CG*T to 74.7% in 5mCG*T. Bypass efficiencies in both situations (for 3′ C and T) increase ∼40% for dG-AF with 5′ 5mC replacing C (compared to the decrement of 90% for dG-AAF). Conversely, when dG-AF is followed by a 3′ purine base (A/G), the bypass efficiency of the lesion decreases from 120.7% in CG*A to 47.5% in 5mCG*A, and from 116.5% in CG*G to 42.1% in 5mCG*G (Figure 2b). These bypass efficiencies of dG-AF (for 3′ A and G) decrease ∼60% with 5′ m5C replacing C (compared to the increment of 40% for dG-AAF).

Next, we conducted the mutagenicity (REAP) assay^35^ on dG-AAF in the 5mC sequence contexts. The results were sequence-dependent. The dG-AAF lesion was not significantly mutagenic in the 3′ pyrimidine (C/T) sequence context, C^#^G*C and C^#^G*T (Figure 3). The average incorporation rate of the correct dG was >96% (e.g., < 4% mutations). An exception was noted with a 14% G to A mutation in the m5CG*C context. The frequency and types of these mutations were consistent with previously reported results.^28^ With a 3′ purine (G/A), we observed a small amount (< 4.4%) of G to C, G to A, and G to T point mutations (Figure 3). It is notable, however, that we additionally observed a significant number of -G deletion mutations (Figure 4b). This contrasted with the no -G deletion with 3′ flanking pyrimidines (Figure 4a). The -G deletion mutation increased from 2.8% in CG*A to 22.9% in 5mCG*A (Figure 4b). In contrast, the level of -G deletion mutation decreased from 45.9% in CG*G to 27.8% in 5mCG*G (Figure 4b). The strong -G deletion mutagenicity of dG-AAF shown here differed significantly from the weakly mutagenic dG-AF adduct and the -GC deletion of dG-AAF in the *Nar*I sequence.^30^

**Figure 3:**
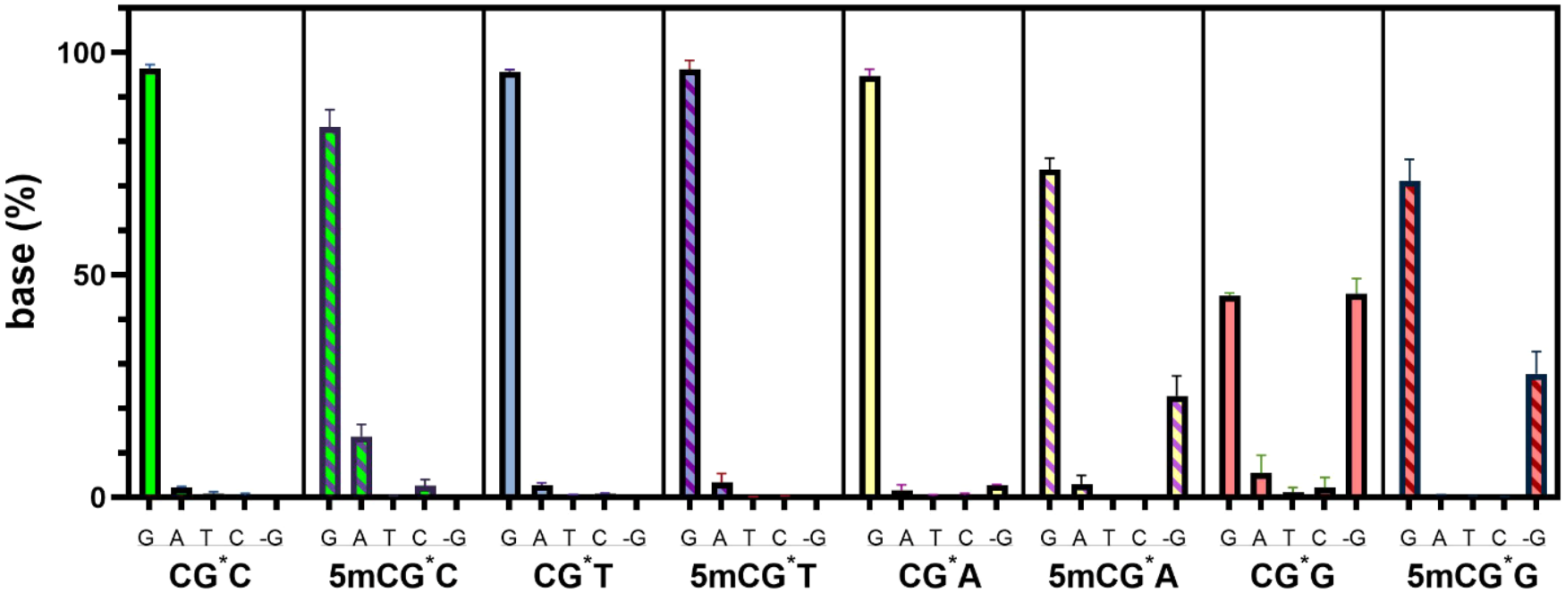
Mutagenicity of dG-AAF in the HK82 *E. coli* strain (AlkB^–^). Data presented as the arithmetic mean (*n* = 3) ± standard deviation (error bar). The central G* base indicated the dG-AAF lesion. Besides the no mutation (G) and point mutations (G to A, G to T, and G to C), we also observed the -G deletion mutation (labeled as -G). *Y*-axis shows the percentage of base composition at the lesion site after replication with 100% representing the total of no mutation (G), point mutations (G to A, G to T, and G to C), and -G deletion mutation. Some of the small error bars are hard to notice; the detailed data are listed in Table S6 in Supporting Information.

**Figure 4:**
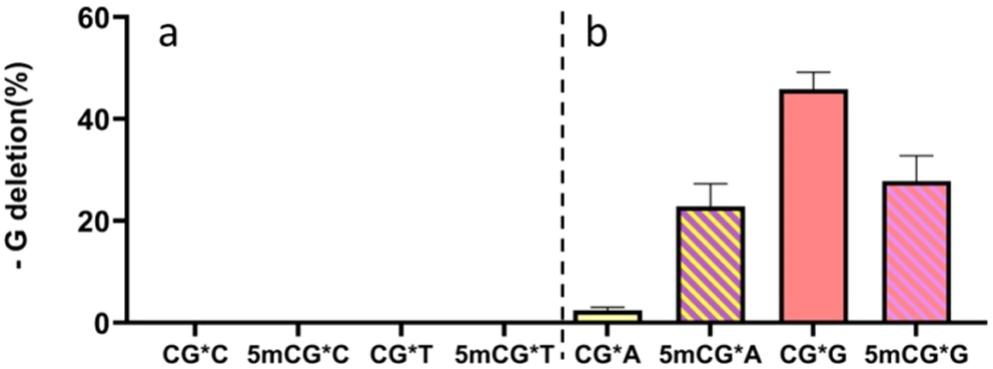
-G deletion mutation of dG-AAF in the HK82 *E. coli* strain (AlkB^–^). The experiments for each sequence context were carried out in triplicate. Data presented as the arithmetic mean ± standard deviation (error bar). The central G* base indicated the dG-AAF lesion. *Y*-axis represents the percentage of -G deletion mutation at the lesion site after replication with 100% representing the total of no mutation (G), point mutations (G to A, G to T, and G to C), and -G deletion mutation. Some of the small error bars are hard to notice; the detailed data are listed in Table S6 in Supporting Information.

## Discussion

In the present work, we first conducted bypass studies of dG-AAF in epigenetically relevant sequence contexts (d­[5′-CTTCTC^#^G*NCCTCATTC-3′], where C^#^ was C or 5-methylcytosine (5mC), G* was dG-AAF, and N was A, T, C, or G). The results showed that adding an epigenetic marker (5′-5mC instead of 5′-C) next to the bulky dG-AAF lesion significantly altered its bypass efficiencies depending on the nature of the 3′ flanking base. In opposite trends, the impact was much more significant with 3′ flanking pyrimidines (∼90% decrease) than with purines (∼40% increase). 3′-C/T in the epigenetically relevant (5′-5mC) sequence appeared to cause a dramatic structural and conformational perturbation, resulting in significant stalling at the replication fork during translesion DNA synthesis. However, the situation was reversed with a 3′-A/G, promoting a stronger bypass.

The dG-AAF and dG-AF lesions are known to exist as a mixture of different types of conformers (such as the S/B/W type) in the DNA duplex (Figure 5a),^33,48,49^ and the resulting conformational heterogeneity is linked to modulating repair and nucleotide insertion efficiencies, respectively. Kinetic studies showed that the impact of the S/B conformation equilibrium at the replication fork is sensitive to the context of the nearby DNA sequence.^49–53^ Unlike the B-conformer, the S/W-conformer likely promotes polymerase stalling because they cannot accommodate the Watson–Crick base pairing required for nucleotide insertion, resulting in a replication block. Therefore, a possible explanation is that S/W conformers of dG-AAF are expected to be predominant in the 3′ pyrimidine contexts in the epigenetically relevant 5mC-containing sequences. In contrast, dG-AAF is likely to adopt the B-conformer in the 3′ purine base context (Figure 2a).

**Figure 5:**
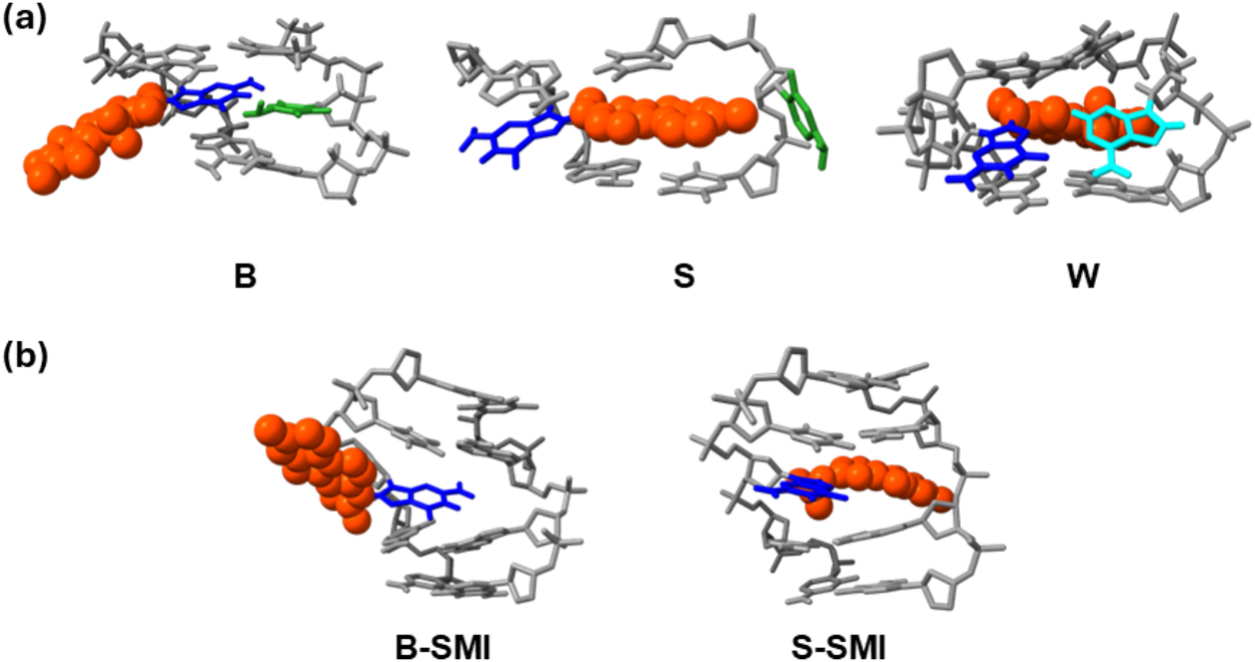
(a) Major groove views from the B-, S-, and W-conformers of dG-AAF: modified G (blue) and complementary C (green) and A (cyan) at the lesion site; *N*-acetyl-2-aminofluorene (orange space-filling).^48^ (b) Major groove views of the B-type- and stacked (S)-slipped mutagenic intermediate (SMI): modified G (blue) and *N*-acetyl-2-aminofluorene (orange space-filling).^32^ These computer modeling-based structures have been generated based on previous solution NMR results.^32,48,54^

We previously conducted a similar bypass study of dG-FAF (fluorine-labeled AF) involving the same set of sequence contexts.^30^ dG-AF is generally bypassed more efficiently than dG-AAF lesions (Figure 2) (∼50% vs ∼10%). These results show that lesion bulkiness could play an essential role because the bulky dG-AAF makes it harder for DNA polymerase to bypass the damage (a decreased bypass efficiency). In contrast, the less bulky dG-AF lesion makes it easier to bypass (increases bypass). Moreover, the 3′ flanking sequence contexts of dG-AF influence bypass efficiency trends opposite to those of the bulky dG-AAF.

Figure 2 illustrates a distinct opposite trend in bypass efficiency for dG-AAF and dG-AF as a function of the C/5mC and 3′-base contexts. Three major factors contribute to this unique trend: lesion bulkiness/conformational heterogeneity, 5′ epigenetic marker 5mC, and 3′-base. dG-AAF is significantly bulkier and conformationally disruptive than dG-AF. As such, the possible S/W-conformeric dG-AAF could significantly alter the active site conformation due to its potential steric clash with the 5′-5mC in the 3′-pyrimidine (C/T) sequence context, contributing to an ∼90% decrease in bypass efficiency. That is not the case for the smaller dG-AF, which showed a moderate increase (40%) in bypass efficiency. Opposite trends were observed for the 3′-purine contexts. We do not know the exact reason for this interesting trend as no current studies on crystals and solutions have been conducted. However, we have previously shown that polymerase stalling at the replication fork is influenced by the S/B/W-conformational complexity, depending on the adduct bulkiness and its unique interactions with surrounding sequence contexts.^33,49^ The situation in the present case is further complicated by the 5mC component. Zhang et. al discovered that 5mC affects the two enantiomers of *anti*-BPDE-dG adduct differently by NMR study. The 10*R*-(−)-*trans*-*anti*-[BP]­dG adduct changes from a minor groove W-type conformation in the unmethylated CpG context to a base-displaced intercalative S-type conformation in the 5mCpG context; whereas the 10*S*-(+)-*trans*-*anti*-[BP]­dG remains W-type conformation regardless of CpG methylation.^55^ Another study shows the crystal structure of DNA methyltransferase DNMT1-DNA complex demonstrates the methyl group of 5-methylcytosine is located in a hydrophobic cave, whereas the cytosine on the opposite strand is looped out.^56^ Also, the effect of methyl in the 5mC epigenetic modification has been shown to decrease DNA backbone flexibility and generally increase the thermal stability of DNA duplexes.^57^ Hence, it may affect the binding of a polymerase, either positively or negatively, depending on the molecular arrangement that occurs in a TLS polymerase’s active site. We postulate that the unique three-way molecular interactions among the bulky lesion, 5′-5mC, and 3′-nucleotide bases play crucial roles in determining the S/B/W heterogeneity, thus determining the polymerase’s stalling and bypassing action.

### Sequence-Dependent Mutational Profile

We observed contrasting patterns of mutations (mutational heterogeneity) for dG-AAF between CG*C/T and CG*A/G. For the former, the predominant type was a small quantity of base substitutions with a single base. Conversely, CG*A/G mutations exhibited a pronounced propensity for −1 frameshift mutations, involving the deletion of a guanine base (Figure 4). We have shown previously that the conformational and thermodynamic stability of bulged-out slipped mutagenic intermediates (SMIs) are critical determinants for the induction of frameshift mutations in the order of −1 deletion > −2 deletion > fully paired > −3 deletion duplexes.^32^ The stacked S-type SMIs are thermodynamically more stable than the conformationally flexible external B-type SMIs (Figure 5b). The conformer ratios of B- and S-SMI appear to cause variations of a frameshift mutation. We postulate that dG-AAF with 3′ flanking purines significantly enhances the formation of S-SMI, resulting in extensive −1 deletions. This is not the case with 3′ pyrimidines, which showed no deletion and presumably exist in mostly the B-SMI conformation. The results show epigenetic marker 5mC demonstrated different impacts on the extent of frameshift mutations (Figure 4b).^32^

In this article, we studied the replication bypass and mutagenicity of dG-AAF next to 5mC. dG-AAF is an adduct to guanine and 5mC is a modification to cytosine; the two adjacent modifications are similar to the situations of tandem or clustered lesions.^58–63^ For example, tandem lesions bearing 5-formyl-2′-deoxyuridine next to a 5′ end 8-oxo-dG or Fapy-dG were discovered; and the tandem lesions are more mutagenic than individual lesions.^64,65^ 2-Nitrofluorene is an IARC-designated Group 2B carcinogen and 2-acetylaminofluorene is an environmental pollutant; both of them can form dG-AAF and dG-AF DNA adducts upon exposure.^26,27^ 5mC is the most abundant DNA modification in eukaryotes; it mainly exists in the CpG sites and accounts for ∼4% of the cytosine residues in the human genome.^5,66,67^ Due to the high abundance of 5mC in the CpG sites, it is likely that the dG-AAF/dG-AF adduct could form at the guanines in the CpG site. Most of the methylations to cytosine are formed immediately after DNA replication. Thus, it is reasonable to speculate that 5mC formation occurs before the neighboring guanine is damaged by dG-AAF and dG-AF.

In summary, adding an epigenetic 5mC marker 5′ to specific DNA locations can drastically influence cells’ efficiencies in bypassing and mutating the bulky dG-AAF DNA lesion. The differences between dG-AAF and dG-AF show that the replication bypass is dependent on (1) lesion-bulkiness (the *N*-acetylated dG-AAF is significantly bulkier than dG-AF); (2) conformational heterogeneity (S/B/W); (3) 3′-sequence context (pyrimidines vs purines); and (4) epigenetic modification (C vs 5mC). The present results demonstrate how bulky dG-AAF adducts alter the cellular replication process. They could be utilized to develop methods for identifying mutations associated with specific mutational signatures or spectra.^68,69^ Furthermore, the present results provide a deeper understanding of the impact of epigenetic markers on DNA replication. Nevertheless, there is still much to be explored. Future research should investigate how lesion-induced conformational complexity, resulting from a 5mC epigenetic marker, impacts DNA translesion synthesis and adduct repair.

## Experimental Procedures

### Oligonucleotides Synthesis and Vector Construction^30,35^

Eight oligonucleotides (d­[5′-CTTCTC^#^G*NCCTCATTC-3′]) (where C^#^ is C or 5mC, G* indicates the lesion position, N is A/G/C/T) were synthesized with site-directed modifications by employing established procedures.^30,34^ The biomimetic activation from 2-nitrofluorene to *N*-hydroyxyesters was used to synthesize DNA-reactive *N*-acetoxy-*N*-(trifluoroacetyl)-2-acetylaminofluorene.^34^ The activated molecule was then diluted in pure alcohol to a concentration of 0.01 mg/mL. This solution was then mixed with a solution of about 50 ODs of unmodified C or 5mC 16mer DNA oligonucleotides in a buffer solution (300 μL of 10 molar sodium citrate, pH 6.0) at 37 °C for 18 to 24 h. The mixtures containing the reaction products were pushed through a syringe filter and then further purified using reverse-phase high-performance liquid chromatography (RP-HPLC) with a Phenomenex Luna C18 column (150 × 10 mm, 5.0 μm; Phenomenex, Torrance, CA) to ensure over 97% purity. In total, eight adducted DNA sequences were created and purified for further analysis. All mass-to-charge ratios of adducted DNA oligonucleotides were verified by LC-ESI-TOF-MS (AB Sciex, ABI4600) (Figures S1–S8 and Table S3). The precise location of the DNA lesion was verified by digesting the DNA with enzymes, followed by MALDI-TOF/MS analysis of the digested fragments to obtain more information about the adduct position (Figures S9–S16 and Table S1).

The 16mer oligonucleotides containing lesions, unmodified controls, and slightly longer 19mer competitor oligonucleotides, whose 5' ends were phosphorylated, annealed with scaffolds, and ligated to form 58mer or 61mer oligonucleotide strands (Figures S17–S18 and Table S2). The formation of lesions containing 58mer oligonucleotides was verified by annealing, restriction enzyme digestion, and LC-ESI-TOF-MS (Figures S19–S27 and Table S4).^30^ These 58mer and 61mer oligonucleotides were then ligated into an M13mp7­(L2) single-stranded vector. The constructed plasmids were purified and recovered; and they were verified by PCR analysis (Figures S28–S29).

### Replication Bypass and Mutagenicity Assays^30,35^

The replication bypass and mutagenicity assays (Figure S30) used ss-M13 DNA vector^35^ containing a dG-AAF adduct. The dG-AAF lesion-containing genomes were mixed with a lesion-free competitor genomes at a 50:1 ratio and transformed into HK82 (AlkB-) *E. coli* strain by electroporation.^37^ The competitor, an internal control, was three nucleotides longer and lacked any lesions. The competitor gauged the replication efficiency and mutagenicity across the sequence of lesions. The M13 phage was then harvested and reamplified in SCS110 cells. The amplified progeny phage DNA was isolated by a QIAprep M13 kit (Qiagen). The DNA was preamplified and amplified in PCR using assay-specific primers, followed by double digestion with XhoI and SphI endonucleases to obtain DNA fragments (20mer/28mer for the adduct sequence and 23mer/31mer for the competitor). Analyses of DNA fragments were performed by LC-ESI-TOF-MS, and all data represented the mean ± the standard deviation (SD) of three independent experiments. The scheme of the procedures is shown in Figures S30 and S31. ESI was conducted using a needle voltage of 4.0 kV in negative ion mode. A heated capillary was set at 350 °C. The nebulizer gas pressure was 35 psi; the heater gas pressure was 20 psi; the curtain gas pressure was 20 psi; the declustering potential was −200 V; and the collision energy was −5 V. Liquid chromatographic separation was achieved by using a C18 column (Acclaim Polar Advantage II C18, 2.1 × 250 mm; 3 μm) at a flow rate of 0.15 mL/min. Solvent A was 500 mM 1,1,1,3,3,3-hexafluoro-2-propanol (HFIP) in water, and solvent B was 500 mM HFIP in 50% methanol at pH 7.0. A solvent gradient was carried out under the following conditions: 25% B for 1 min, 25 to 50% of B over 2 min, 50 to 75% B over 20 min, 75 to 100% B over 1 min, 100% B for 10 min, 80 to 25% B over 1 min, and 25% B over 10 min. The LC column oven was set at 35 °C during the entire running time. To quantify the lesion bypass, the ratio between the intensities of the 20 or 28 mer and 23 mer fragments was determined and normalized to the ratio obtained from the experiments employing an unmodified “G” control, considered as 100% bypass (Figure 2 and Table S5). Mutagenicity was analyzed based on the mass spectrometry (MS) results (Figure 3 and Table S6), and mutation frequency was calculated by dividing the signal of individual mutated oligonucleotides by the total signals.

## Supplementary Material



## Notes

ESI-TOF analyses of oligonucleotides. MALDI-TOF mass spectra of digestion of lesion-containing oligonucleotides; diagrams of experimental procedures and results; tables of Mass spectrometry analyses, and bypass efficiency and mutagenicity (PDF)
