# Supplementary material for: Conformation-Dependent Lesion Bypass and Mutagenicity of Bulky 2‑Acetylaminofluorene-Guanine DNA Adduct in Epigenetically Relevant Sequence Contexts
Source: Chem Res Toxicol. 2025 Jul 4;38(8):1336–43. doi: 10.1021/acs.chemrestox.5c00055 (PMC12422539; doi:10.1021/acs.chemrestox.5c00055)
Supplement: Supplementary file 1 [file tx5c00055_si_001.pdf]

SUPPORTING INFORMATION

Conformation-dependent lesion bypass and mutagenicity of bulky 2-acetylaminofluorene-guanine DNA adduct in epigenetically-relevant sequence contexts

Yi-Tzai Chen, Rui Qi, Ang Cai, Bongsup P. Cho*, and Deyu Li*

Department of Biomedical and Pharmaceutical Sciences, College of Pharmacy, University of Rhode Island, Kingston, RI, 02881, United States of America

* To whom correspondence should be addressed. Tel: +1 (401) 874-9361 (DL);

+1 (401) 874-5024 (BPC). Email: deyuli@uri.edu (DL); bcho@uri.edu (BPC).

Table of Contents

Figure S1. ESI-TOF analysis of 16mer oligo containing CG*C (G*=dG-AAF).

Figure S2. ESI-TOF analysis of 16mer oligo containing 5mCG*C (G*=dG-AAF).

Figure S3. ESI-TOF analysis of 16mer oligo containing CG*T (G*=dG-AAF).

Figure S4. ESI-TOF analysis of 16mer oligo containing 5mCG*T (G*=dG-AAF).

Figure S5. ESI-TOF analysis of 16mer oligo containing CG*A (G*=dG-AAF).

Figure S6. ESI-TOF analysis of 16mer oligo containing 5mCG*A (G*=dG-AAF).

Figure S7. ESI-TOF analysis of 16mer oligo containing CG*G (G*=dG-AAF).

Figure S8. ESI-TOF analysis of 16mer oligo containing 5mCG*G (G*=dG-AAF).

Figure S9. MALDI-TOF mass spectra of digestion of 16mer oligo containing CG*C (G*=dG-AAF).

Figure S10. MALDI-TOF mass spectra of digestion of 16mer oligo containing 5mCG*C (G*=dG-AAF).

Figure S11. MALDI-TOF mass spectra of digestion of 16mer oligo containing CG*T (G*=dG-AAF).

Figure S12. MALDI-TOF mass spectra of digestion of 16mer oligo containing 5mCG*T (G*=dG-AAF).

Figure S13. MALDI-TOF mass spectra of digestion of 16mer oligo containing CG*A (G*=dG-AAF).

Figure S14. MALDI-TOF mass spectra of digestion of 16mer oligo containing 5mCG*A (G*=dG-AAF).

Figure S15. MALDI-TOF mass spectra of digestion of 16mer oligo containing CG*G (G*=dG-AAF).

Figure S16. MALDI-TOF mass spectra of digestion of 16mer oligo containing 5mCG*C (G*=dG-AAF).

Figure S17. Diagram of construction of the 58mer lesion containing oligonucleotide using the CG*C sequence as an example (G*=dG-AAF).

Figure S18. Denaturing urea polyacrylamide gel of the 58mer lesion containing oligonucleotide using the CG*A sequence as an example (G*=dG-AAF).

Figure S19. Diagram of LC-TOF-MS Identification of the 58mer lesion containing oligonucleotide using the CG*C sequence as an example (G*=dG-AAF).

Figure S20. ESI-TOF analysis of 20mer oligo containing CG*C (G*=dG-AAF).

Figure S21. ESI-TOF analysis of 20mer oligo containing 5mCG*C (G*=dG-AAF).

Figure S22. ESI-TOF analysis of 20mer oligo containing CG*T(G*=dG-AAF).

Figure S23. ESI-TOF analysis of 20mer oligo containing 5mCG*T (G*=dG-AAF).

Figure S24. ESI-TOF analysis of 20mer oligo containing CG*A (G*=dG-AAF).

Figure S25. ESI-TOF analysis of 20mer oligo containing 5mCG*A (G*=dG-AAF).

Figure S26. ESI-TOF analysis of 20mer oligo containing 5mCG*G (G*=dG-AAF).

Figure S27. ESI-TOF analysis of 20mer oligo containing 5mCG*G (G*=dG-AAF).

Figure S28. Diagram of PCR analysis for lesion containing M13 genome using the CG*C sequence as an example (G*=dG-AAF).

Figure S29. Diagram of 15% polyacrylamide gel of PCR products of lesion containing M13 genome using the CG*A sequence (G*=dG-AAF) as an example.

Figure S30. Diagram of the REAP & CRAB procedures using $C^{\#}$G*N as an illustration (G*=dG-AAF).

Figure S31. Typical LC-TOF-MS spectra of the REAP and CRAB samples.

Figure S32. Bypass efficiency and mutagenicity of εA in HK82 cells (AlkB-).

Table S1. Calculated and observed MW and m/z measured by MALDI-TOF (G*=dG-AAF).

Table S2. List of oligonucleotides and primer sequences (5′→3′) used for the REAP and CRAB assays (G*=dG-AAF).

Table S3. Calculated and observed monoisotopic MW and m/z values of modified oligonucleotides (G*=dG-AAF).

Table S4. Calculated and observed monoisotopic MW and m/z values of modified oligonucleotides after digestion (G*=dG-AAF).

Table S5. Bypass efficiency (CRAB assay) of dG-AAF/AF in HK82 (AlkB-) E. coli cells (G*=dG-AAF/AF).

Table S6. Mutagenicity (REAP assay) of dG-AAF in HK82 (AlkB-) E. coli cells (G*=dG-AAF).

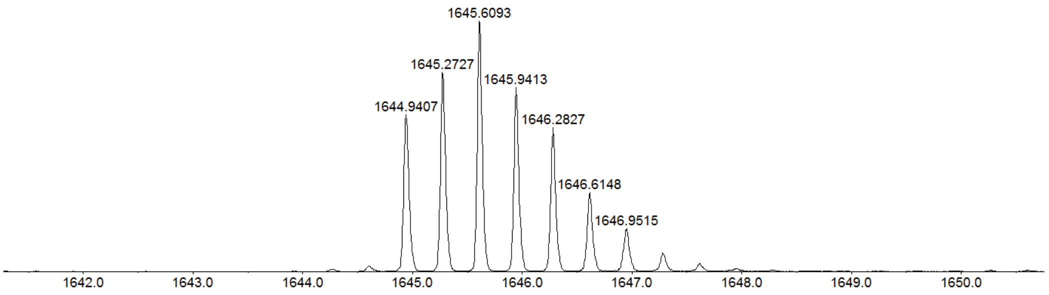

Figure S1. ESI-TOF analysis of 16mer oligo containing CG*C (G*=dG-AAF). Data represent the −3 charge envelope.

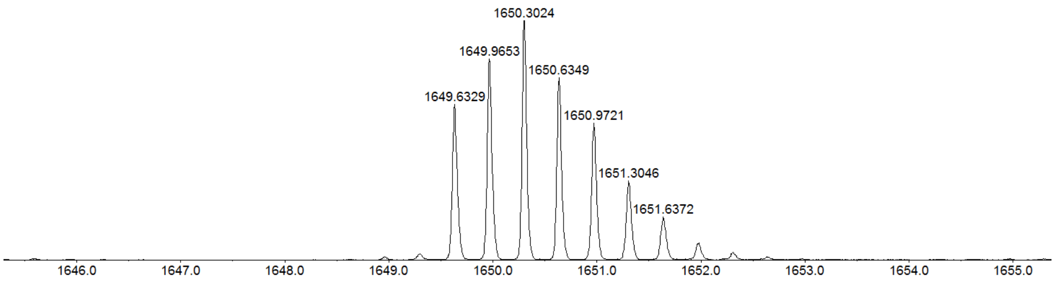

Figure S2. ESI-TOF analysis of 16mer oligo containing 5mCG*C (G*=dG-AAF). Data represent the −3 charge envelope.

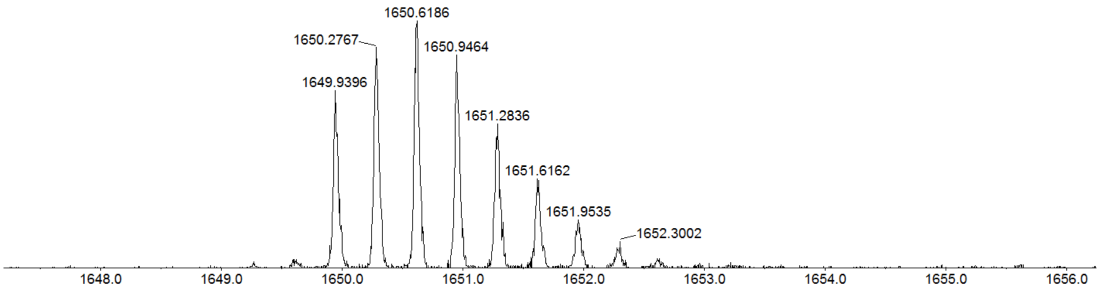

Figure S3. ESI-TOF analysis of 16mer oligo containing CG*T (G*=dG-AAF). Data represent the −3 charge envelope.

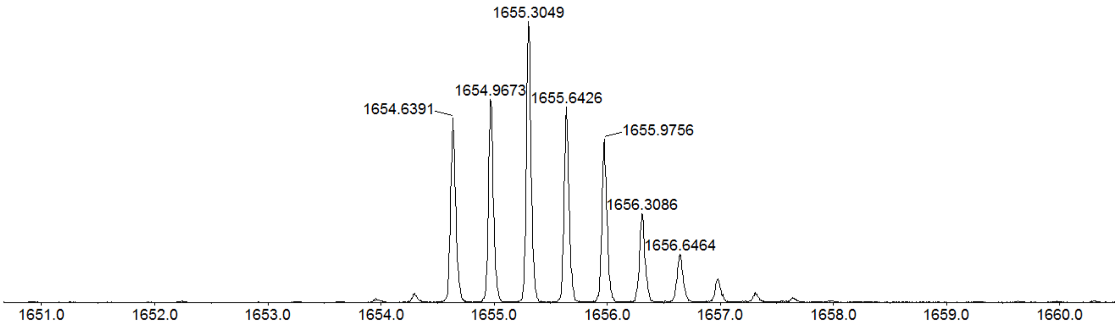

Figure S4. ESI-TOF analysis of 16mer oligo containing 5mCG*T (G*=dG-AAF). Data represent the −3 charge envelope.

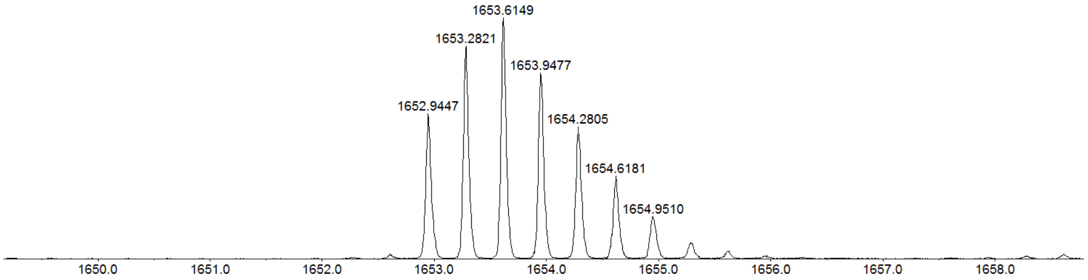

Figure S5. ESI-TOF analysis of 16mer oligo containing CG*A (G*=dG-AAF). Data represent the −3 charge envelope.

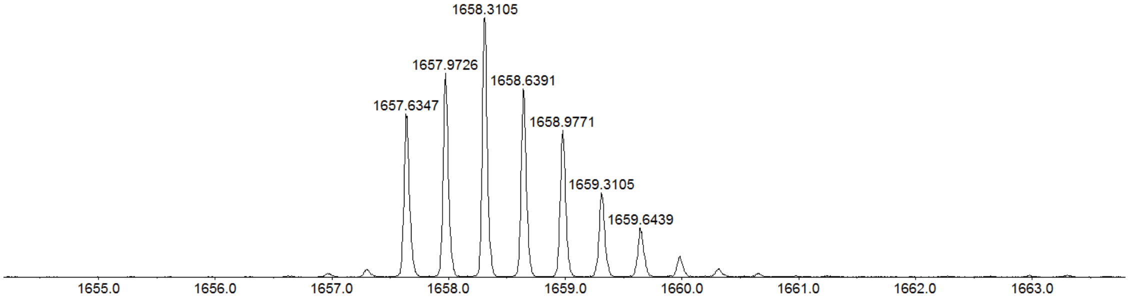

Figure S6. ESI-TOF analysis of 16mer oligo containing 5mCG*A (G*=dG-AAF). Data represent the −3 charge envelope.

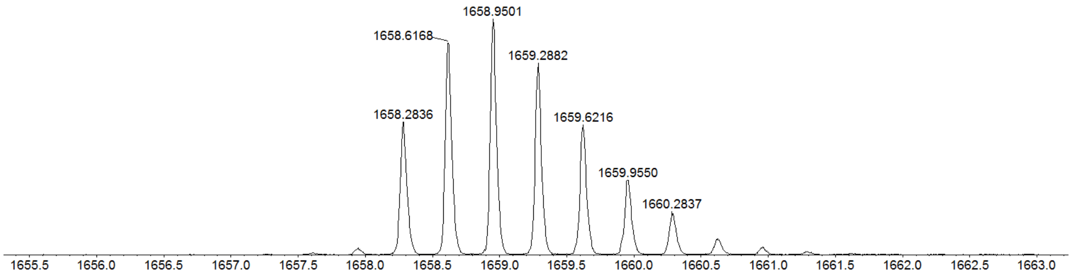

Figure S7. ESI-TOF analysis of 16mer oligo containing CG*G (G*=dG-AAF). Data represent the −3 charge envelope.

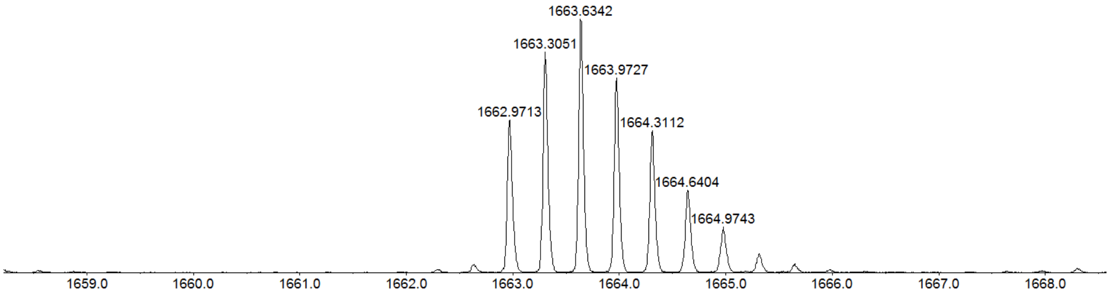

Figure S8. ESI-TOF analysis of 16mer oligo containing 5mCG*G (G*=dG-AAF). Data represent the −3 charge envelope.

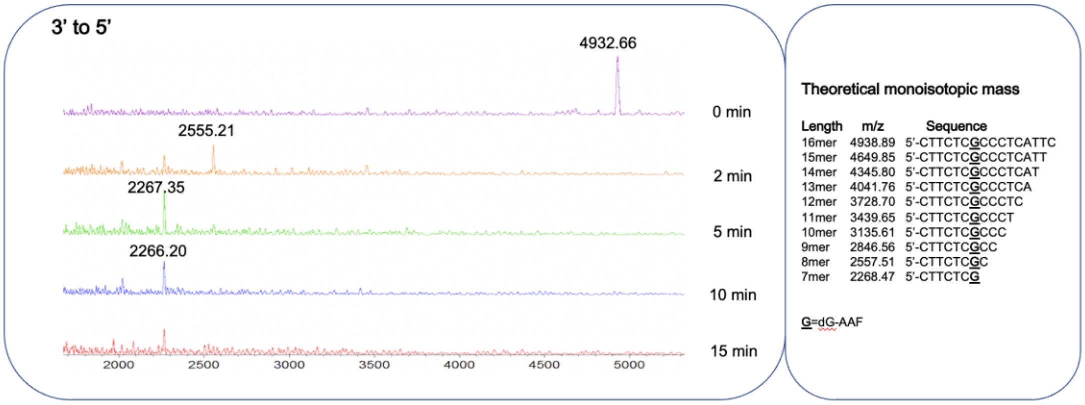

Figure S9. MALDI-TOF mass spectra of digestion of 16mer oligo containing CG*C (G*=dG-AAF). 3′→5′ Exonuclease digestions (SVP enzyme) in Reflectron mode are shown at various time intervals. Insets provide theoretical m/z of the corresponding fragments.

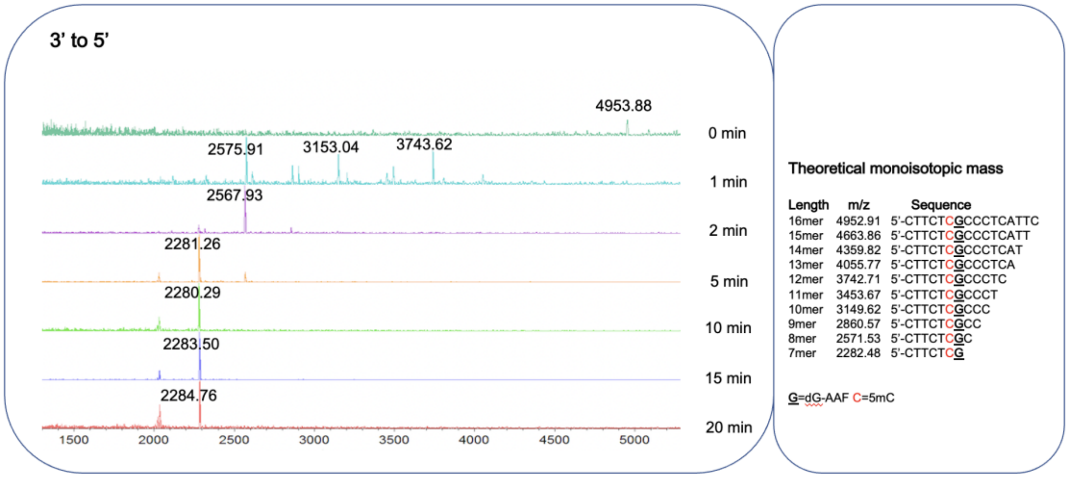

Figure S10. MALDI-TOF mass spectra of digestion of 16mer oligo containing 5mCG*C (G*=dG-AAF). 3′→5′ Exonuclease digestions (SVP enzyme) in Reflectron mode are shown at various time intervals. Insets provide theoretical m/z of the corresponding fragments.

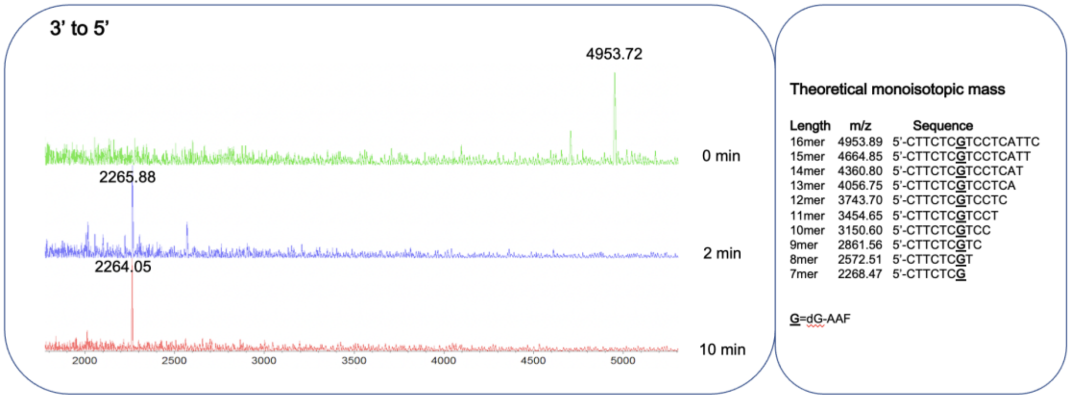

Figure S11. MALDI-TOF mass spectra of digestion of 16mer oligo containing CG*T (G*=dG-AAF). 3′→5′ Exonuclease digestions (SVP enzyme) in Reflectron mode are shown at various time intervals. Insets provide theoretical m/z of the corresponding fragments.

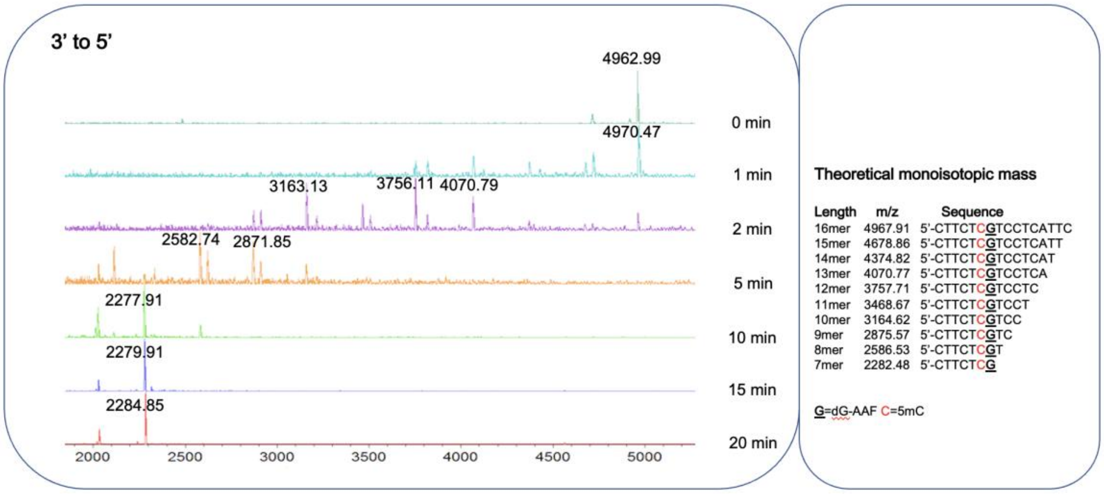

Figure S12. MALDI-TOF mass spectra of digestion of 16mer oligo containing 5mCG*T (G*=dG-AAF). 3′→5′ Exonuclease digestions (SVP enzyme) in Reflectron mode are shown at various time intervals. Insets provide theoretical m/z of the corresponding fragments.

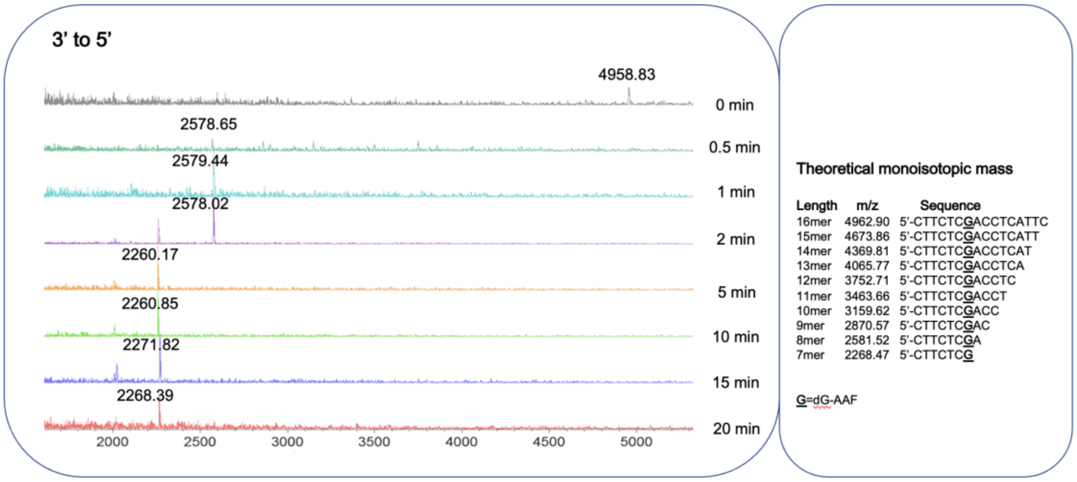

Figure S13. MALDI-TOF mass spectra of digestion of 16mer oligo containing CG*A (G*=dG-AAF). 3′→5′ Exonuclease digestions (SVP enzyme) in Reflectron mode are shown at various time intervals. Insets provide theoretical m/z of the corresponding fragments.

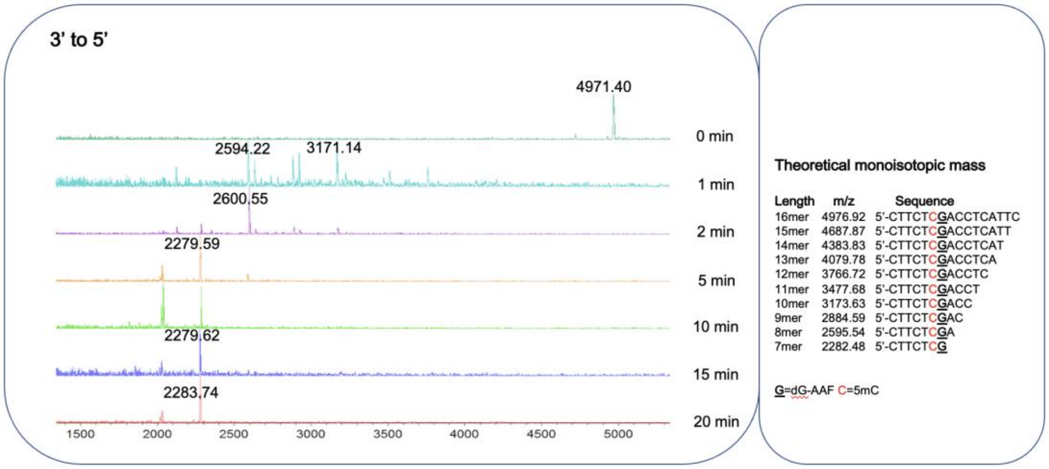

Figure S14. MALDI-TOF mass spectra of digestion of 16mer oligo containing 5mCG*A (G*=dG-AAF). 3′→5′ Exonuclease digestions (SVP enzyme) in Reflectron mode are shown at various time intervals. Insets provide theoretical m/z of the corresponding fragments.

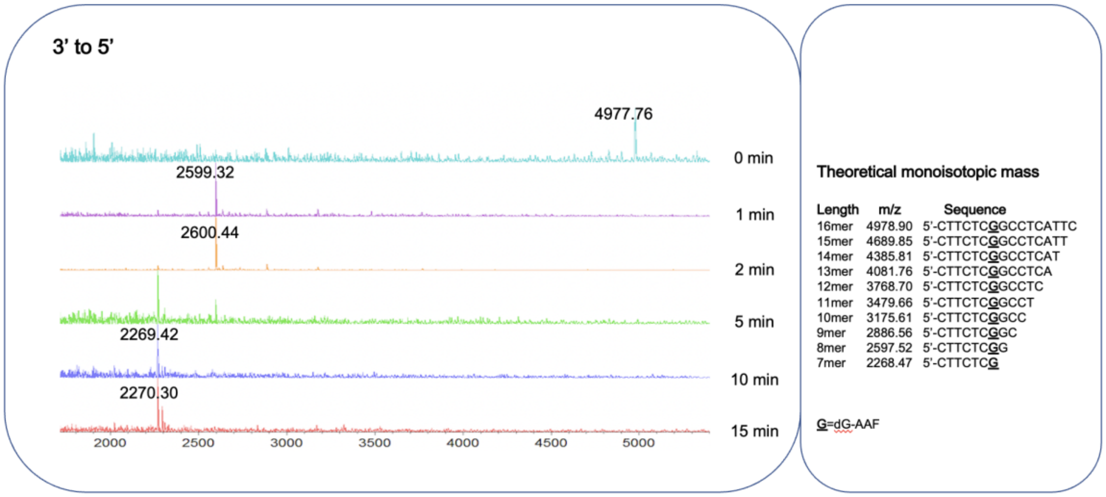

Figure S15. MALDI-TOF mass spectra of digestion of 16mer oligo containing CG*G (G*=dG-AAF). 3′→5′ Exonuclease digestions (SVP enzyme) in Reflectron mode are shown at various time intervals. Insets provide theoretical m/z of the corresponding fragments.

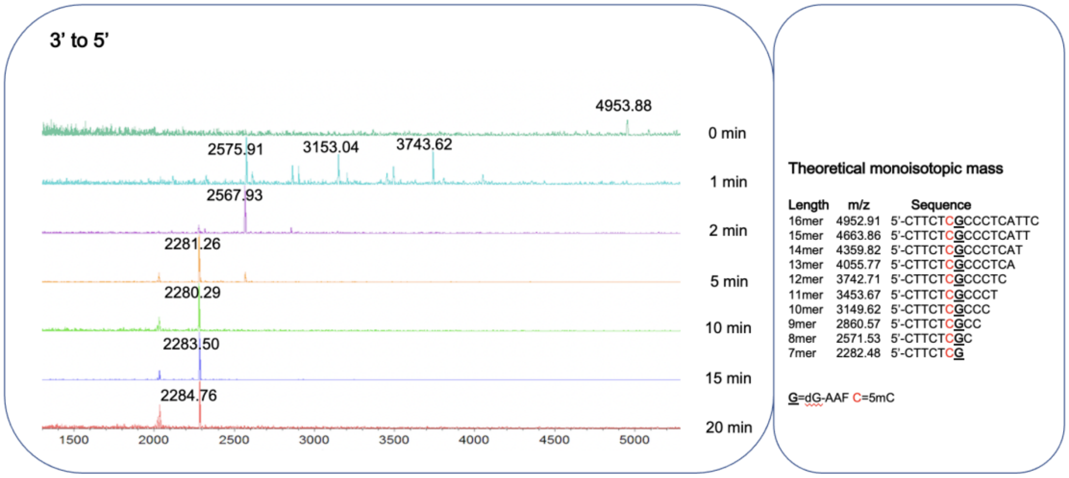

Figure S16. MALDI-TOF mass spectra of digestion of 16mer oligo containing 5mCG*C (G*=dG-AAF). 3′→5′ Exonuclease digestions (SVP enzyme) in Reflectron mode are shown at various time intervals. Insets provide theoretical m/z of the corresponding fragments.

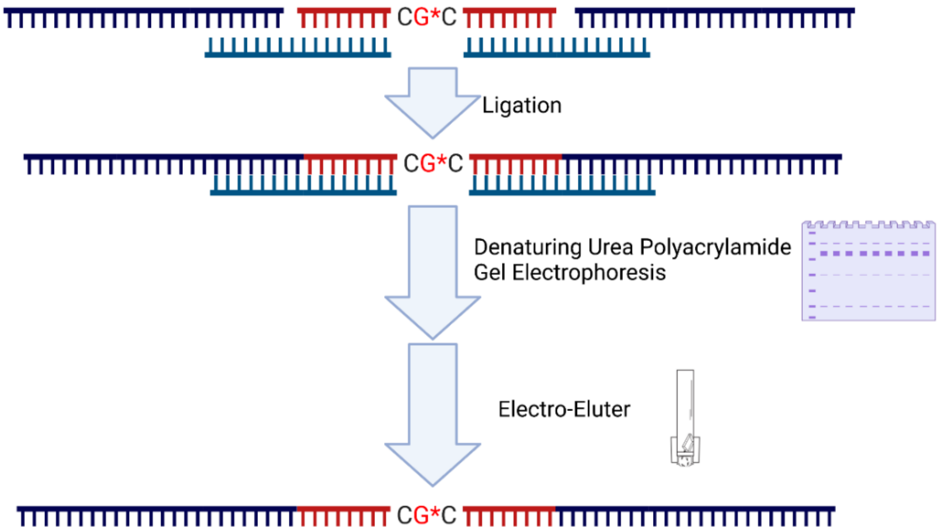

Figure S17. Diagram of construction of the 58mer lesion containing oligonucleotide using the CG*C sequence as an example (G*=dG-AAF).

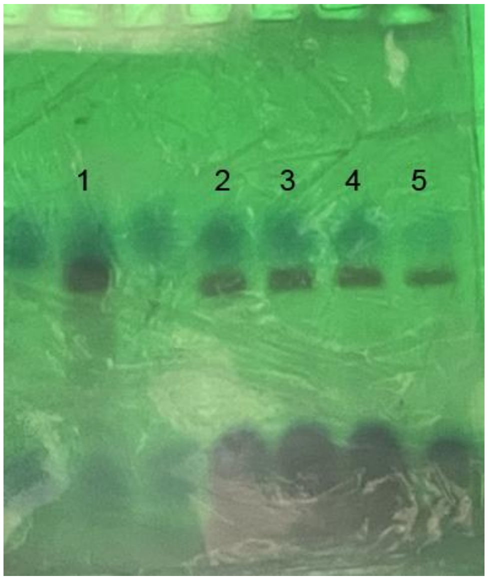

Figure S18. Denaturing urea polyacrylamide gel of the 58mer lesion containing oligonucleotide using the CG*A sequence as an example (G*=dG-AAF). Lane 1, 58mer Control. Lane 2-5, 58mer CG*A ligation product.

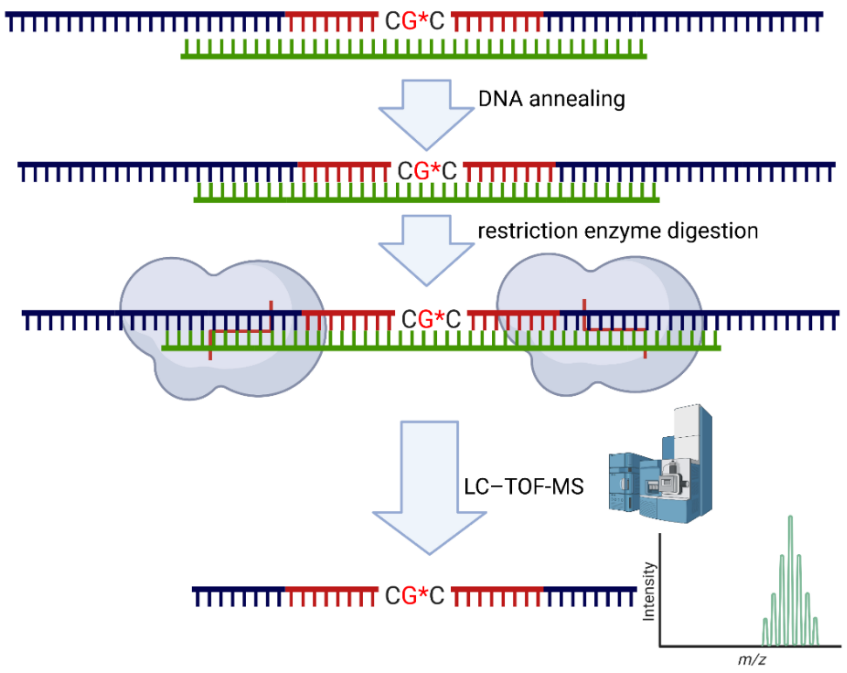

Figure S19. Diagram of LC-TOF-MS Identification of the 58mer lesion containing oligonucleotide using the CG*C sequence as an example (G*=dG-AAF).

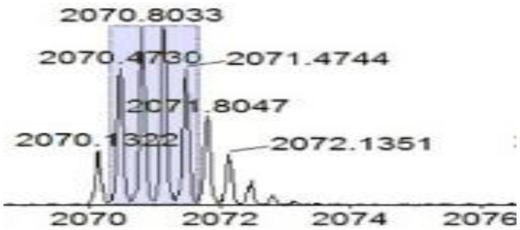

Figure S20. ESI-TOF analysis of 20mer oligo containing CG*C (G*=dG-AAF). Data represent the −3 charge envelope.

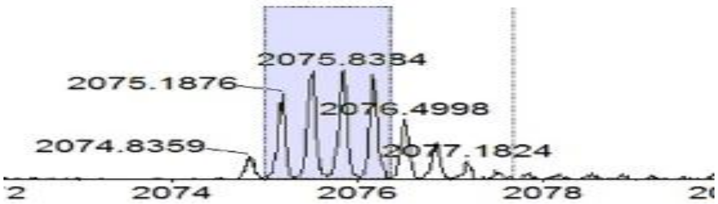

Figure S21. ESI-TOF analysis of 20mer oligo containing 5mCG*C (G*=dG-AAF). Data represent the −3 charge envelope.

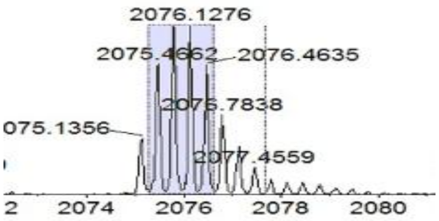

Figure S22. ESI-TOF analysis of 20mer oligo containing CG*T(G*=dG-AAF). Data represent the −3 charge envelope.

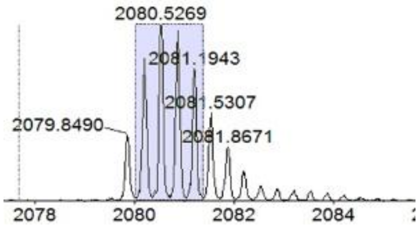

Figure S23. ESI-TOF analysis of 20mer oligo containing 5mCG*T (G*=dG-AAF). Data represent the −3 charge envelope.

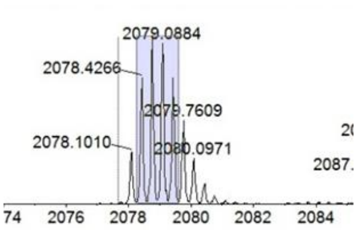

Figure S24. ESI-TOF analysis of 20mer oligo containing CG*A (G*=dG-AAF). Data represent the −3 charge envelope.

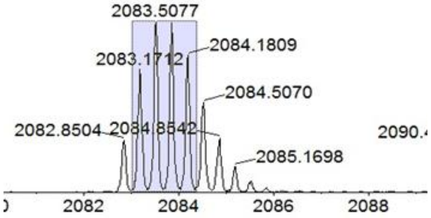

Figure S25. ESI-TOF analysis of 20mer oligo containing 5mCG*A (G*=dG-AAF). Data represent the −3 charge envelope.

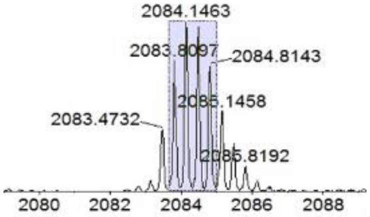

Figure S26. ESI-TOF analysis of 20mer oligo containing 5mCG*G (G*=dG-AAF). Data represent the −3 charge envelope.

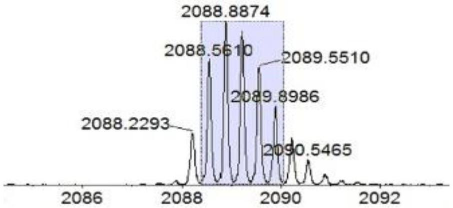

Figure S27. ESI-TOF analysis of 20mer oligo containing 5mCG*G (G*=dG-AAF). Data represent the −3 charge envelope.

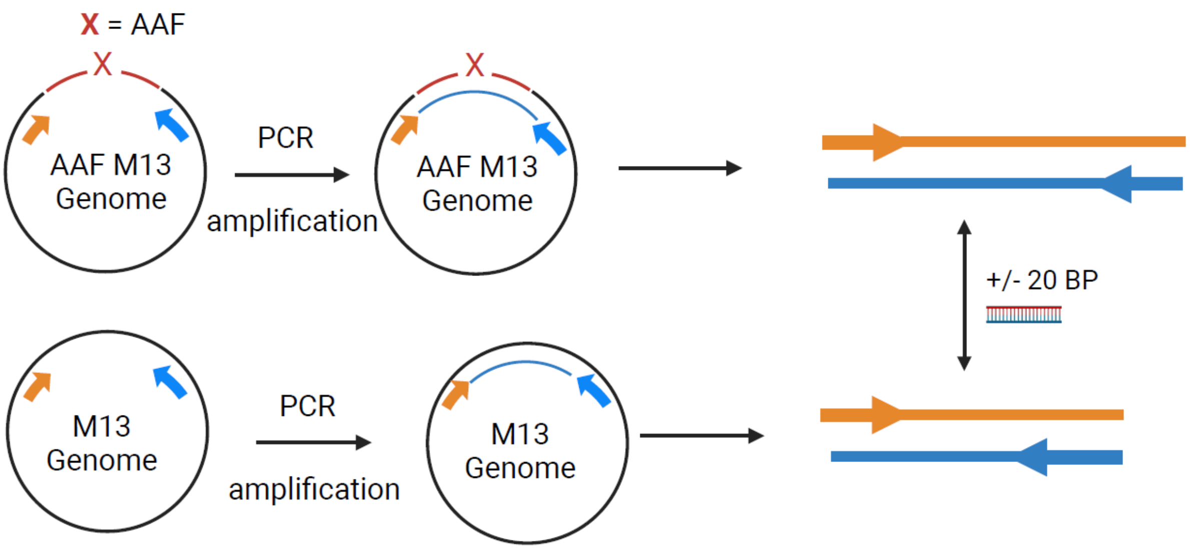

Figure S28. Diagram of PCR analysis for lesion containing M13 genome using the CG*C sequence as an example (G*=dG-AAF).

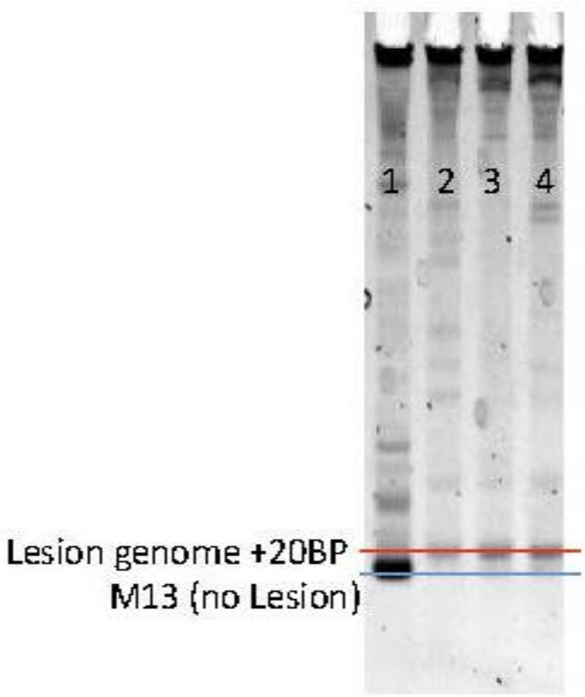

Figure S29. Diagram of 15% polyacrylamide gel of PCR products of lesion containing M13 genome using the CG*A sequence (G*=dG-AAF) as an example. Lane 1, M13 genome Control. Lane 2-4, M13 genome contains 58mer CG*A ligation product (G*=dG-AAF).

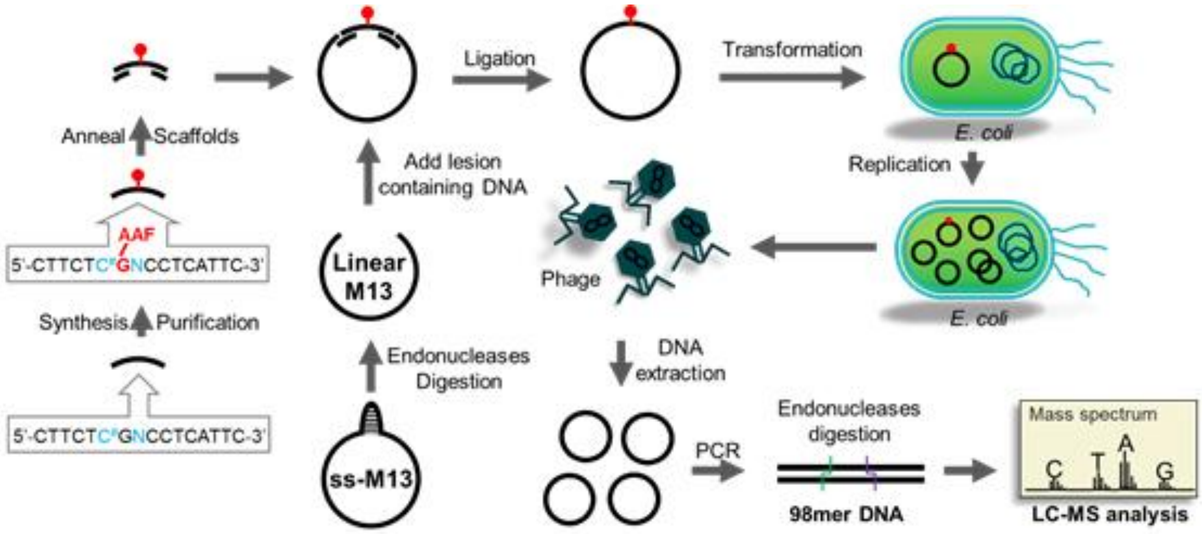

$$\text{Bypass} = {}^{\text{Lesion signal/competitor signal}}/_{\text{control signal/competitor signal}}$$

$$\text{Mutation frequency} = {}^{\text{mutation signal}}/_{\text{all base components signal}}$$

Figure S30. Diagram of the REAP & CRAB procedures using $C^{\#}G^{*}N$ as an illustration (G^{*}=dG-AAF).

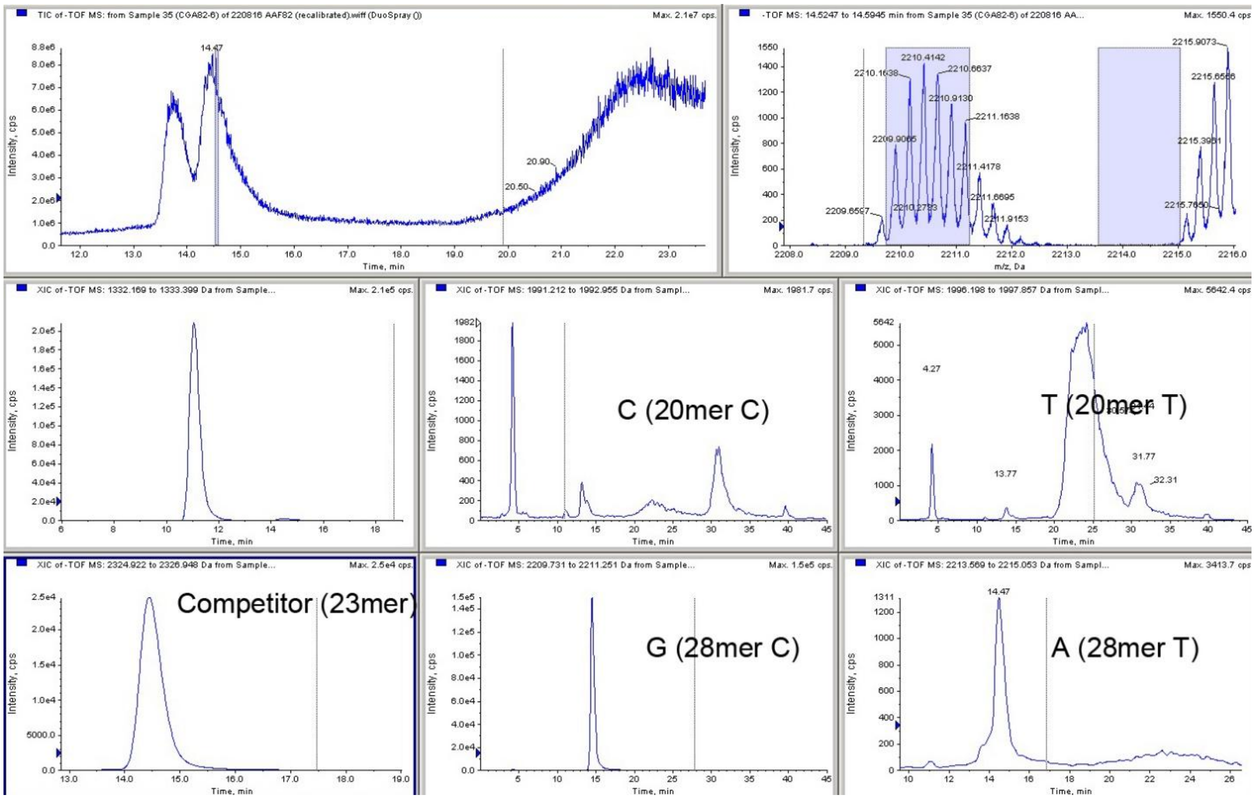

Figure S31. Typical LC-TOF-MS spectra of the REAP and CRAB samples. The C (20mer C), T (20mer T) and competitor (23mer) are monitored at the -3 charge state. The G (28mer C) and A (28mer T) are monitored at -4 charge state.

Lesion mutational specificity and frequency *in vivo*

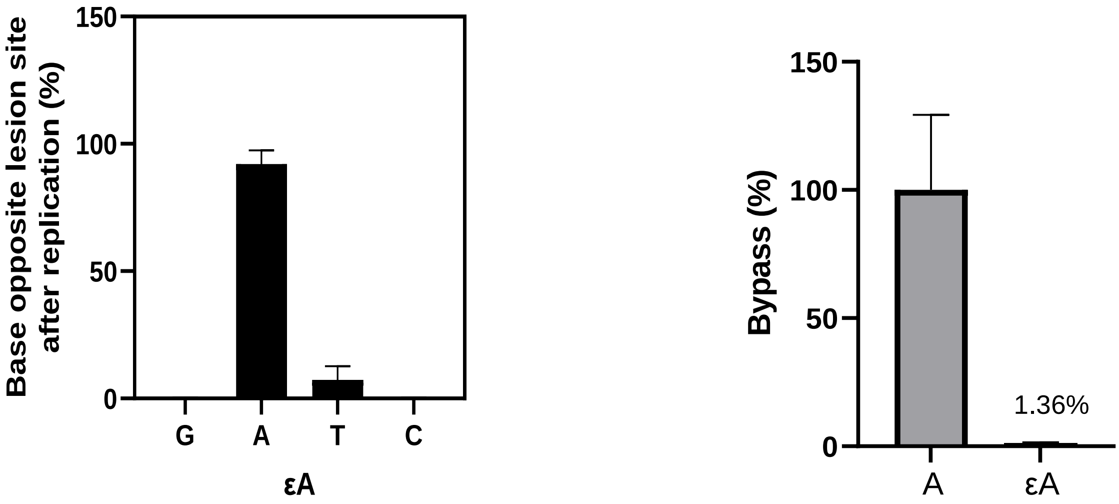

Figure S32. Bypass efficiency and mutagenicity of εA in HK82 cells (AlkB-). Left panel shows mutation patterns of εA and right panel shows the bypass efficiency of εA.

Table S1. Calculated and observed MW and m/z measured by MALDI-TOF (G*=dG-AAF).

Sequence	Calculated Mass		Observed Mass	
	Full Length (Da)	Digested (Da)	Full Length (Da)	Digested (Da)
CG*C	4938.89	2268.47	4932.66	2266.20
5mCG*C	4952.91	2282.48	4953.88	2280.50
CG*T	4953.89	2268.47	4953.72	2265.88
5mCG*T	4967.91	2282.48	4970.47	2279.91
CG*A	4962.90	2268.47	4458.83	2268.39
5mCG*A	4976.92	2282.48	4971.40	2279.62
CG*G	4978.90	2268.47	4977.76	2269.42
5mCG*G	4952.91	2282.48	4953.88	2281.26

Table S2. List of oligonucleotides and primer sequences (5′→3′) used for the REAP and CRAB assays (G*=dG-AAF).

CGC	CGC Control	CTTCTCGCCCTCATTC
	CGC Control 3' end oligo	TACCGTCGAGACGCGCATGCA
	CGC Control 5' end oligo	TCTCGAGTGCATCGTCAGCAC
	CG*C	CTTCTCG*CCCTCATTC
	CG*C 3' end oligo	TACCGTCGACTCGCGCATGCA
	CG*C 5' end oligo	TCTCGAGTGACTCGTCAGCAC
	5mCG*C	CTTCT5mCG*CCCTCATTC
	5mCG*C 3' end oligo	TACCGTCGCATCGCGCATGCA
	5mCG*C 5' end oligo	TCTCGAGTGATGCGTCAGCAC
	Complementary	CACTCGAGAGAATGAGGGCGAGAAGTGCATGCGCG
CGT	CGT Control	CTTCTCGTCCTCATTC
	CGT Control 3' end oligo	TACCGTCGAACCGCGCATGCA
	CGT Control 5' end oligo	TCTCGAGTGACTCGTCAGCAC
	CG*T	CTTCTCG*TCCTCATTC
	CG*T 3' end oligo	TACCGTCGTTCCGCGCATGCA
	CG*T 5' end oligo	TCTCGAGTGATGCGTCAGCAC
	5mCG*T	CTTCT5mCG*TCCTCATTC
	5mCG*T 3' end oligo	TACCGTCGCGGCGCGCATGCA
	5mCG*T 5' end oligo	TCTCGAGTGATGCGTCAGCAC
	Complementary	CACTCGAGAGAATGAGGACGAGAAGTGCATGCGCG
CGA	CGA Control	CTTCTCGACCTCATTC
	CGA Control 3' end oligo	TACCGTCGGTTCGCGCATGCA
	CGA Control 5' end oligo	TCTCGAGTGCATCGTCAGCAC
	CG*A	CTTCTCG*ACCTCATTC
	CG*A 3' end oligo	TACCGTCGCGGCGCGCATGCA
	CG*A 5' end oligo	TCTCGAGTGCATCGTCAGCAC

	5mCG*A	CTTCT5mCG*ACCTCATTC
	5mCG*A<br>3’ end oligo	TACCGTCGAGACGCGCATGCA
	5mCG*A<br>5’ end oligo	TCTCGAGTGATGCGTCAGCAC
	Complementary	GCGCGTACGTGAAGAGCTGGAGTAAGAGAGCTCAC
CGG	CGG Control	CTTCTCGGCCTCATTC
	CGG Control<br>3’ end oligo	TACCGTCGTCACGCGCATGCA
	CGG Control<br>5’ end oligo	TCTCGAGTGACTCGTCAGCAC
	CG*G	CTTCTCG*GCCTCATTC
	CG*G<br>3’ end oligo	TACCGTCGGGCCGCGCATGCA
	CG*G<br>5’ end oligo	TCTCGAGTGATGCGTCAGCAC
	5mCG*G	CTTCTC5mG*GCCTCATTC
	5mCG*G<br>3’ end oligo	TACCGTCGACTCGCGCATGCA
	5mCG*G<br>5’ end oligo	TCTCGAGTGATGCGTCAGCAC
	Complementary	GCGCGTACGTGAAGAGCCGGAGTAAGAGAGCTCAC
AAF Competitor	19mer	CTTCTTGACCTCATTCTAG
	19mer<br>3’ end oligo	TACCGTCGCTACGCGCATGCA
	19mer<br>5’ end oligo	TCTCGAGTGTGTCGTCAGCAC
εA	εA Control	GAAGACCTAGGCGTCC
	εA Control<br>3’ end oligo	TACCGTCGTTCCGCGCATGCA
	εA Control<br>5’ end oligo	TCTCGAGTGAACCGTCAGCAC
	εA	GAAGACCTA*GGCGTCC
	εA<br>3’ end oligo	TACCGTCGTAGCGCGCATGCA
	εA<br>5’ end oligo	TCTCGAGTGAACCGTCAGCAC
	εA Competitor	GAAGACCTAGGCGTCCTAG
	εA Competitor<br>3’ end oligo	TACCGTCGATGCGCGCATGCA
	εA Competitor<br>5’ end oligo	TCTCGAGTGAACCGTCAGCAC
Scaffold I		AGAAGTGCATGCGCG
Scaffold II		CACTCGAGAGAATGAGG

190bp primer Forward		TTGTGTGGAATTGTGAGCGG
190bp primer Reverse		TGCAAGGCGATTAAGTTGGG
230bp primer Forward		CACCCCAGGCTTTACACTTT
230bp primer Reverse		GCAAGGCGATTAAGTTGGGTAA
MS Forward primer		CGCCAGGGTTTTCCCAGTCACGAC
MS Reverse primer		AGCGGATAACAATTTCACACAGGA

Table S3. Calculated and observed monoisotopic MW and m/z values of modified oligonucleotides (G*=dG-AAF).

5’-CTTCTC#G*NCCTCATTC-3’	MW (calculated) of neutral species	m/z (calculated) -3 charge peak	m/z (observed) -3 charge peak
16mer CG*C	4937.8857	1644.9541	1644.9407
16mer CG*T	4952.8853	1649.9540	1649.9396
16mer CG*A	4961.8969	1652.9578	1652.9447
16mer CG*G	4977.8918	1658.2894	1658.2836
16mer 5mCG*C	4951.9013	1649.6260	1649.6329
16mer 5mCG*T	4966.9010	1654.6258	1654.6391
16mer 5mCG*A	4975.9126	1657.6297	1657.6347
16mer 5mCG*G	4991.9075	1662.9613	1662.9713

Table S4. Calculated and observed monoisotopic MW and m/z values of modified oligonucleotides after digestion (G*=dG-AAF).

	MW (calculated) of neutral species	m/z (calculated) -3 charge peak	m/z (observed) -3 charge peak
20mer CG*C	6133.0821	2070.0083	2070.1332
20mer CG*T	6148.0817	2075.0082	2075.1356
20mer CG*A	6157.0933	2078.0121	2078.1010
20mer CG*G	6173.0882	2083.3437	2083.4732
20mer 5mCG*C	6147.0977	2074.6802	2074.8359
20mer 5mCG*T	6162.0974	2079.6801	2079.8490
20mer 5mCG*A	6171.1090	2082.6839	2082.8504
20mer 5mCG*G	6187.1039	2088.0156	2088.2293

Table S5. Bypass efficiency (CRAB assay) of dG-AAF/AF in HK82 (AlkB-) E. coli cells (G*=dG-AAF/AF). The data of dG-AF are extracted from "Crisalli, A. M., Chen, Y.-T., Cai, A., Li, D. & Cho, B. P. Conformation-dependent lesion bypass of bulky arylamine-dG adducts generated from 2-nitrofluorene in epigenetic sequence contexts. Nucleic Acids Res. 51, 12043–12053 (2023)."

dG-AAF

N=	C	T	A	G
CG*N	8.6±0.7	9.3±0.9	6.1±0.6	5.2±0.4
5mCG*N	0.2±0.1	1.0±0.2	8.8±0.4	7.9±1.2

dG-AF

N=	C	T	A	G
CG*N	54.7±6.6	55.7±10.0	120.7±5.0	116.5±2.6
5mCG*N	79.7±4.1	74.7±12.9	47.5±2.1	42.1±2.0

Table S6. Mutagenicity (REAP assay) of dG-AAF in HK82 (AlkB-) E. coli cells (G*=dG-AAF).

N.D.: not detected.

N=	G	A	T	C	-G
CG*C	96.3±1.0	2.2±0.3	0.9±0.4	0.6±0.4	N.D.
5mCG*C	83.3±3.9	13.8±2.7	0.3±0.2	2.7±1.4	N.D.
CG*T	95.6±0.5	2.8±0.5	0.7±0.1	0.9±0.1	N.D.
5mCG*T	96.1±2.1	3.4±2.0	0.1±0.1	0.3±0.1	N.D.
CG*A	94.7±1.6	1.7±1.2	0.4±0.3	0.6±0.4	2.8±0.2
5mCG*A	73.8±2.5	3.1±1.9	0.1±0.1	0.3±0.0	22.9±4.5
CG*G	45.4±0.6	5.5±4.0	1.2±1.1	2.2±2.4	45.9±3.3
5mCG*G	71.1±5.0	0.6±0.1	0.4±0.0	0.2±0.0	27.8±5.0
